# Voltage‐dependent potassium currents expressed in *Xenopus laevis* oocytes after injection of mRNA isolated from trophozoites of *Giardia lamblia* (strain Portland‐1)

**DOI:** 10.1002/phy2.186

**Published:** 2013-12-29

**Authors:** Arturo Ponce, Enedina Jimenez‐Cardoso, Leticia Eligio‐Garcia

**Affiliations:** 1 Department of Physiology, Biophysics and Neurosciences, Center for Research and Advanced Studies IPN, México City, Mexico; 2 Parasitology Research Laboratory, Children Hospital of México “Federico Gomez”, Mexico City, México

**Keywords:** *Giardia lamblia*, K currents, voltage clamp, *Xenopus* oocytes

## Abstract

Despite its importance as a health problem issue, almost nothing is known about the membrane physiology of *Giardia lamblia* and practically there exist no information so far regarding the variety and properties of ion channels that this protozoan parasite possesses. To address this subject we resorted to an indirect method, consisting in the injection of mRNA and further characterization of ion currents in *Xenopus* oocytes. In this work, we show that oocytes injected with mRNA isolated from cultured trophozoites of *G. lamblia*, strain Portland‐1 express novel potassium currents that appear over the second day after injection and show time‐ and voltage‐dependent activation followed by a slow inactivation. They start activating at −90 mV, with V_1/2_ of −30 mV; its time constant of activation (at +60 mV) is 0.11 sec, whereas that of inactivation is 1.92 sec, V_1/2_ = −44.6 mV. Such K currents were effectively blocked by K channel blockers TEA and 4AP, as well as Ba^2+^, quinine, quinidine, charybdotoxin, dendrotoxin‐1, capsaicin, margatoxin, and diltiazem. These results suggest that such currents are the result of expression of Giardia′s voltage‐gated K channels heterologously expressed in *Xenopus laevis* oocytes.

## Abstract (precis)

In this work, we show that mRNA injection of Giardia´s trophozoites induces expression of novel ion currents in *Xenopus leavis* oocytes. The magnitude as well as the biophysical and pharmacological properties of these currents suggests that they are the result of exogenous potassium channels newly incorporated into the membrane of frog's oocytes.

## Introduction

*Giardia lamblia* is a flagellated protozoan that thrives in the intestinal lumen of several mammal species, including human beings. Even though it can be harmless to its host, most frequently causes mild to moderate gastrointestinal or allergic diseases. Its life cycle consists of two alternate stages: A freely motile trophozoite and a dormant, environmentally resistant cyst. This parasitic *Diplomonad* is broadly spread worldwide and is a major source of diarrheal diseases among human population, with an estimate of over 250 million cases per year, mainly in developing countries. Despite its importance as a health problem almost nothing is currently known about its cellular and molecular physiology (Adam 1991; Gillin et al. 1996).

Ion channels are essential components of the plasma membrane that regulate the flux of ions (sodium, potassium, calcium, and chloride) between the cell and its environment or among different intracellular compartments. (Alberts et al. 2002; Kew and Davies 2009). They comprise a vastly diverse type of protein complexes that, besides their largely known role on excitable cells and on the maintenance of the resting membrane potential (Hille 2001; Hille and Catterall 1999) have been found to be involved in many physiologically important processes including: regulation of cell cycle and proliferation (Kunzelmann 2005; Blackiston et al. 2009), cell migration (Schwab et al. 2012), and cell volume regulation (Strange et al. 1996; Ponce et al. 2012) among many others. It is thought now that ion channels are present in all types of cells, from bacteria to higher metazoan species, yet there are no reports to date describing what types of ion channels are expressed in *Giardia*, notwithstanding the fact that its genome is already available and it is partially annotated (Aurrecoechea et al. 2009). As a first contribution to this subject we have resorted to an indirect experimental approach: The heterologous expression of ion channels in oocytes of *Xenopus laevis*, a South African clawed frog. After injection with sample mRNA, these cells eventually synthesize exogenous channels whose properties can be assayed by electrophysiological and pharmacological criterions (Dascal 1987; Theodoulou and Miller 1995a). This method has shown to be highly valuable to probe ion channels and receptors from distinct mammal tissues and from species as diverse as viruses (Kang et al. 2004); bacteria (Maksaev and Haswell 2011); plants (Theodoulou and Miller [Bibr b42][Bibr b43]); Invertebrates such as *Caenorhabditis* (Martínez‐Torres and Miledi 2006), *Aplysia* (Pfaffinger et al. 1991), and *Taenia* (Ponce et al. 2008). Furthermore, ion channel properties from protozoan parasites such as *Entamoeba* (Delgadillo et al. 2000, 2002 and Salas‐Casas et al. 2006) and *Leishmania* (Figarella et al. 2007 and Lagos et al. 2007) had been described using this method.

In this work, we focused on the study of potassium (K) channels, the largest and most diverse group of ion channels (Coetzee et al. 1999). We show that injection of mRNA isolated from trophozoites of *G. lamblia* induces expression of exogenous K currents in *Xenopus laevis* oocytes and describe their biophysical and pharmacological properties.

## Materials and Methods

### Isolation and purification of mRNA from *Giardia lamblia*

Trophozoites of *Giardia lamblia*, strain Portland‐1 (ATCC‐30888) were axenically cultured in TYI‐S‐33 media at 37°C for 72 h (Farthing et al. 1983). Total RNA was extracted and purified from 1 × 10^6^ trophozoites with the Ambion^®^ RNAqueous^®^ Kit (Cat. Number AM1912, Life Technologies, Carlsbad, CA). Messenger RNA was further purified with the Dynabeads mRNA Purification Kit (Cat 61006, Ambion) following the procedures described by the manufacturer. Prior to injection mRNA was resuspended in RNAse‐free water at 1 *μ*g/*μ*L. Degraded mRNA, which was made for control purposes, was obtained by incubating mRNA with 1 *μ*g/*μ*L Rnase A (12091‐039, Invitrogen) at 37°C for 30 min.

### Dissection of *Xenopus* oocytes and injection

General procedures were as described elsewhere (Goldin 1992; Ponce et al. 2008). To obtain oocytes, adult female frogs were anesthetized by immersion in an induction tank containing 0.1% Tricaine (MS‐222, Sigma‐Aldrich A‐5040, St. Louis, MO) for 15 min. After ventral incision ovarian lobes were partially removed and immersed in a Petri dish containing saline solution OR2 (see below for composition) and kept at 18°C for 8 h in a low‐temperature incubator (VWR 2005), next oocytes were manually detached of the ovarian lobes. The follicular layer was removed by incubation with 2 mg/mL collagenase type 1 (C9891, Sigma) in free‐calcium ND96 solution with gentle agitation at room temperature. Released oocytes were rinsed twice with ND96 solution and kept overnight at 18°C. The next day, mature (stage IV) oocytes were sorted and deposited on a Petri dish containing ND96, to be injected manually using a microinjection pipet (Manual Oocyte Microinjection Pipet, cat No. 3‐000‐510‐X, Drummond Scientific Company, Broomall, PA) mounted on a mechanic micromanipulator (MN153, Narishige Scientific Instruments, Tokyo, Japan). The injection process was monitored with a stereoscopic microscope (SMZ‐1500, Nikon Corporation, Shinjuku, Tokyo, Japan). Oocytes were individually injected with 50 nL of any of water or degraded or intact mRNA (1 *μ*g/*μ*L). After injection, oocytes were incubated at 16°C in ND96 supplemented with gentamycin (5 mg/mL) until recording of ion currents. The trial was reviewed and approved by the Internal Committee of the Center for Research and Advanced Studies (CICUAL), in accordance with the Mexican Official Normativity for Ethical Care and Management of laboratory animals (NOM‐062‐ZOO).

### Electrophysiology

Ion currents were recorded from oocytes using the standard two‐electrode voltage clamp technique (Stühmer 1992; Schwarz and Rettinger 2003). The setup consisted of a TEV 200 amplifier (Dagan Corporation, Minneapolis, MN) connected to a PC computer through an analog‐digital converter (Digidata 1322A, Molecular Devices LLC, Sunnyvale, CA). Voltage clamp‐stimulating protocols and acquisition of currents were made with the Clampex module of the software suite pclamp 6.0 (Molecular Devices LLC). Both current injecting and voltage sensing microelectrodes were made from borosilicate glass capillary tubing, with inner filament (GT‐15, Warner Instruments, Hamden, CT), pulled to a tip resistance of 1–2 mol/L Ω on a Brown–Flaming type puller (P87, Sutter Instruments, Novato, CA), and backfilled with 3 mol/L KCl. Reference electrodes were connected to a recording chamber through glass bridges filled with 200 m mol/L NaCl in 2% agarose (A9539, Sigma‐Aldrich). Oocytes were placed in a chamber filled with saline recording solution (whose composition is described below) and impaled with both electrodes. This procedure was verified optically and electrically, by monitoring a sudden change in the membrane potential. After impalement, voltage was held at −80 mV. The external bathing solution was continuously perfused by gravity at a rate of 5 mL/min. Distinct protocols of voltage were used as detailed below (see results). Current signals were processed online with a low‐pass filter, at a cutoff frequency of 200 Hz, and sampled at 34.5 Hz. All assays were performed at room temperature.

### Solutions and chemicals

An OR2 solution, that was used throughout dissection, consisted of 82.5 mmol/L NaCl, 2.5 mmol/L KCl, 1.0 mmol/L CaCl_2_, 1.0 mmol/L MgCl_2_, 1.0 mmol/L Na_2_HPO_4_, 5.0 mmol/L HEPES, pH 7.8. Standard bath solution ND96 was used during injection and contained 96 mmol/L NaCl, 2 mmol/L KCl, 1.8 mmol/L CaCl_2_, 1 mmol/L MgCl_2_, 5 mmol/L HEPES, pH 7.4. During incubation oocytes were maintained with ND96 solution complemented with 6 mmol/L sodium pyruvate (P5287, Sigma Aldrich) and 100 *μ*g/mL gentamicin (G1397, Sigma‐Aldrich). For recording of K^+^ currents, endogenous Ca^2+^‐dependent Cl^−^ currents were excluded by substitution of Cl^−^ with gluconate (glnt‐) in the bath medium, also CaCl_2_ was withdrawn and 5 mmol/L EGTA was added; therefore, the composition of the recording bath medium was 96 mmol/L Na‐glnt, 2 mmol/L K‐glnt, 5 mmol/L EGTA, 1 mmol/L Mg‐glnt_2_, 5 mmol/L HEPES, pH 7.4. Additionally, it was complemented with channel blockers DIDS 500 *μ*mol/L, and 9‐AC 1 mmol/L. For K^+^ selectivity assays the composition of the recording solution was modified by replacing 20 and 70 mmol/L Na‐glnt by the same amount of K‐glnt. All chemical and reagents, including K channel blockers, were from Sigma‐Aldrich.

### Analysis of data

All signal processing procedures, such as filtering and compensation of linear components (capacitive and leak currents), as well as measurement of ion current magnitude were made offline with the clampfit module of the Pclamp software suite (Molecular Devices). Mathematical procedures such as transformation of data and curve fitting were made with Sigmaplot 12 (Systat Software, Bangalore, Karnataka, India). Statistical calculations and tests were made with Microsoft Excel (Office 2010). Results are reported as mean ± SEM (number of cases). Differences among treatment groups were analyzed using Student's *t*‐test, assuming unequal variances, with Null difference among groups. Multiple comparisons were made with one‐way analysis of variance, followed by Student's *t*‐test corrected for multiple comparisons (Fisher's exact test). A *P* < 0.05 was taken as a minimal criterion to consider Null hypothesis rejection.

## Results

### Injection of mRNA from *Giardia lamblia* produces hyperpolarization on *Xenopus* oocytes

**Figure 1.**
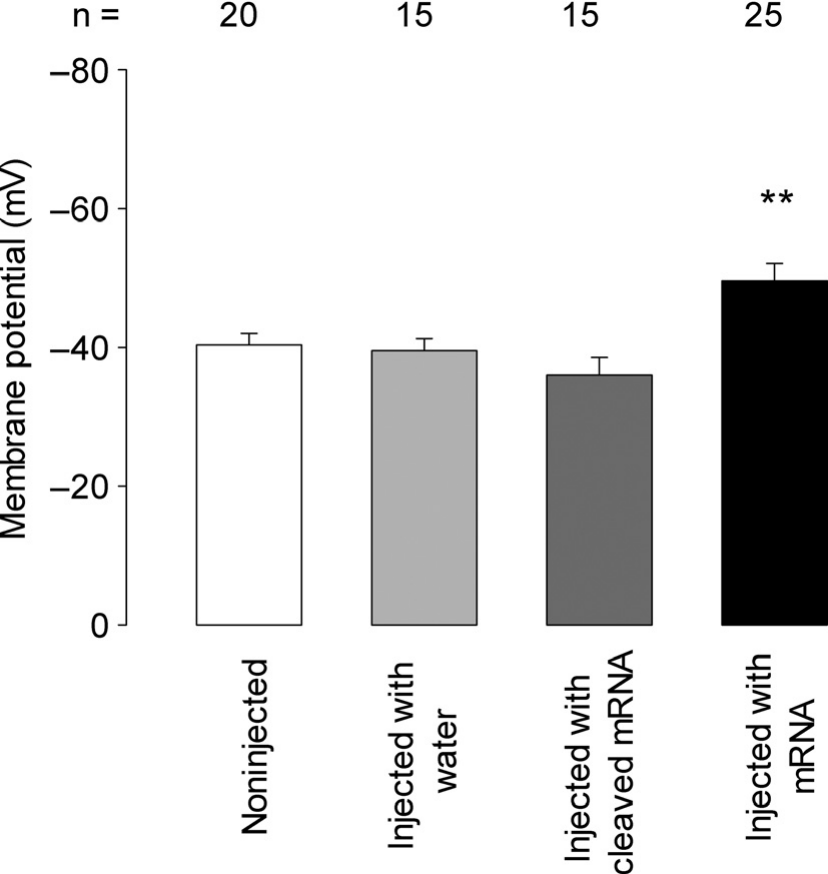
Effect of *Giardia lamblia *mRNA injection on the membrane potential (Em) of *Xenopus* oocytes. Bar length indicates the mean value (±SE) of membrane potential of individual mature oocytes that were either noninjected or injected with water, degraded mRNA or intact mRNA. Negative values are shown upward for convenience. Em measurements were made on oocytes 3 days after injection. **P < 0.005 for a *t*‐test, comparing noninjected versus injected with mRNA. Numbers above bars indicate number of cases. Multiple comparisons test (Tukey) showed no statistically significant difference among noninjected values with neither injected with water nor injected with degraded mRNA.

First, we sought to determine if injection of mRNA, isolated from *G. lamblia* trophozoites, in *Xenopus* oocytes induces changes to the membrane potential (Em), as this would suggest changes on the properties of the oocyte′s membrane as a result of expression of exogenous ion channels. For this purpose, we measured Em, with intracellular microelectrodes, from mature de‐folliculated oocytes and compared it among different experimental conditions. As Fig. 1 shows, noninjected oocytes had a mean value of −40.4 ± 1.7 mV (*n* = 20), whereas that of oocytes injected with mRNA was −49.6 ± 2.5 mV (*n* = 25), which was significantly more negative (*P* < 0.005, t‐test). In contrast, oocytes that were injected with either water or degraded mRNA, as negative controls, had mean values of −39.5 ± 1.7 mV (*n* = 15) and −36.0 ± 2.4 mV (*n* = 15), which were not significantly distinct from noninjected, but they were from injected with mRNA (*P* < 0.005, ANOVA one‐way and Tukey, pairwise comparisons with 95% IC). Accordingly, these results indicate that oocytes hyperpolarize as a consequence of *G. lamblia*′s mRNA injection.

### Injection of mRNA isolated from *Giardia lamblia* induces expression of exogenous ion currents in oocytes

**Figure 2.**
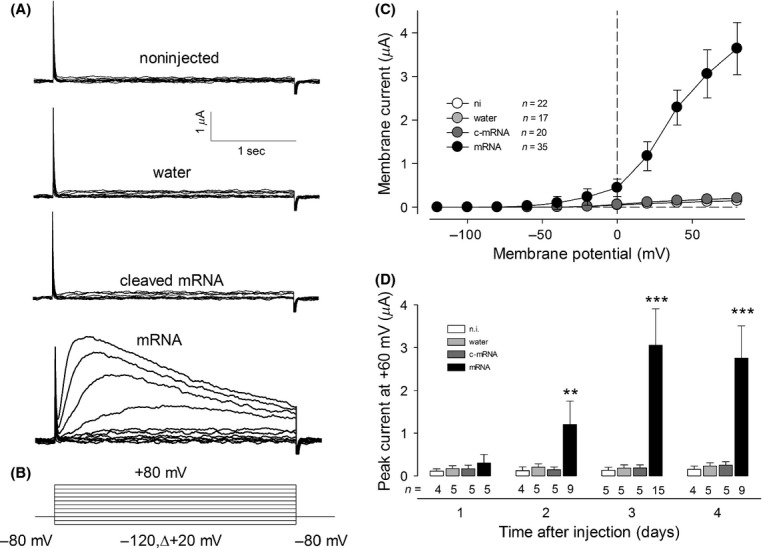
Expression of exogenous ion currents by injection of oocytes with mRNA isolated from *Giardia lamblia*. (A) Representative series of traces of current obtained from oocytes under the distinct experimental conditions indicated on the legends, they all are depicted at the same magnitude indicated in the scale bar at the top. Currents were obtained in response to series of squared voltage pulses that as shown in (B) were from −120 up to +80 mV in steps of 20 mV from a holding level of −80 mV. (C) Relationship between the maximal current amplitude and the test voltage from oocytes injected with intact mRNA as well as noninjected or mock injected. Dots indicate the average value (±SE) of the number of individual oocytes assayed that the inset indicates. (D) Time course of the expression of currents after days of injection. Bar length indicates the mean value of peak current in response to a voltage pulse of +60 mV.

The hyperpolarization induced by injection of mRNA could be in part due to the expression of novel ion pathways in the plasma membrane. To test this possibility we recorded ion currents of injected and noninjected oocytes with the electrophysiological technique of two‐electrode voltage clamp. In this work, we focused on the characterization of K^+^ currents, therefore anion currents were excluded by substitution on the bathing media, of chloride by gluconate, an impermeant organic anion, along with the addition of blockers of Cl channels DIDS 500 *μ*mol/L and 9‐AC 1 mmol/L (Lu et al. 1990), also Ca^2+^‐dependent Cl^−^ currents were excluded by withdrawing of CaCl_2_ and addition of 5 mmol/L EGTA to the bath media. Figure 2A shows (*top to bottom*) representative series of ion current recordings obtained from oocytes from each of four distinct treatments: (1) noninjected or injected with (2) water or (3) intact or (4) degraded *Giardia′s* mRNA. All these recordings were made 3 days after injections and were produced by the same voltage protocol (depicted in Fig. 2B) that consisted of series of squared test voltage pulses, for 3 sec each (from −120 to +80 in steps of +20 mV) from a holding voltage of −80 mV; a lapse of 60 sec was allowed between pulses. Figure 2C shows the relationship between the amplitude of current and the test voltage of all four treatments. Ion currents recordings from noninjected oocytes were barely distinguishable from background over the entire range of voltage tested, with a mean amplitude at +80 mV (*I*_80)_ of 0.15 ± 0.07 *μ*A (*n* = 20), similar currents were recorded form oocytes injected with water as a negative control (*I*_80_ = 0.21 ± 0.08 *μ*A, *n* = 15). In contrast, oocytes injected with intact Giardia's mRNA had currents noticeably different from those obtained from noninjected or injected with water: They were barely distinguishable from background in the more negative range of voltage but, from about −60 mV, started increasing its amplitude with depolarizing voltage pulses. Its magnitude at +80 mV, *I*_80_ = 3.6 ± 0.6 *μ*A (*n* = 25) is far larger than the control groups. The time course of these currents is also distinct; they gradually increased its magnitude over time, until reaching a peak value and then stably decreased. These results suggest therefore that injection of mRNA isolated from *G. lamblia* elicits expression of novel ion currents in *Xenopus* oocytes. This conclusion is further supported by the fact that such currents were not observed in oocytes injected with degraded mRNA that were rather similar to those obtained from noninjected oocytes (*I*_80_ = 0.21 ± 0.08 *μ*A, *n* = 15).

Our next goal was to determine how these currents develop in time after injection, for this purpose we measured the amplitude of the membrane current, in response to a test pulse of +60 mV, every day up to 4 days, from oocytes that were noninjected or injected with water, degraded mRNA or intact mRNA. As Fig. 2D shows, after 1 day of injection, there is no difference statistically significant among those groups (*P* > 0.05, ANOVA), however, from the second day this difference become statistically significant (*P* < 0.01, ANOVA), as the amplitude of ion currents from oocytes injected with mRNA gets larger than those from noninjected or injected with water or degraded mRNA, which remains unchanged throughout this time. From this study we learned that the average magnitude of ion currents from oocytes injected with mRNA reached a maximum value at the third day after injection (3.1 ± 0.8 *μ*A), for this reason, further assays were made at this time.

### Ion currents induced in *Xenopus* oocytes by injection of *Giardia lamblia*′s mRNA are potassium selective

**Figure 3.**
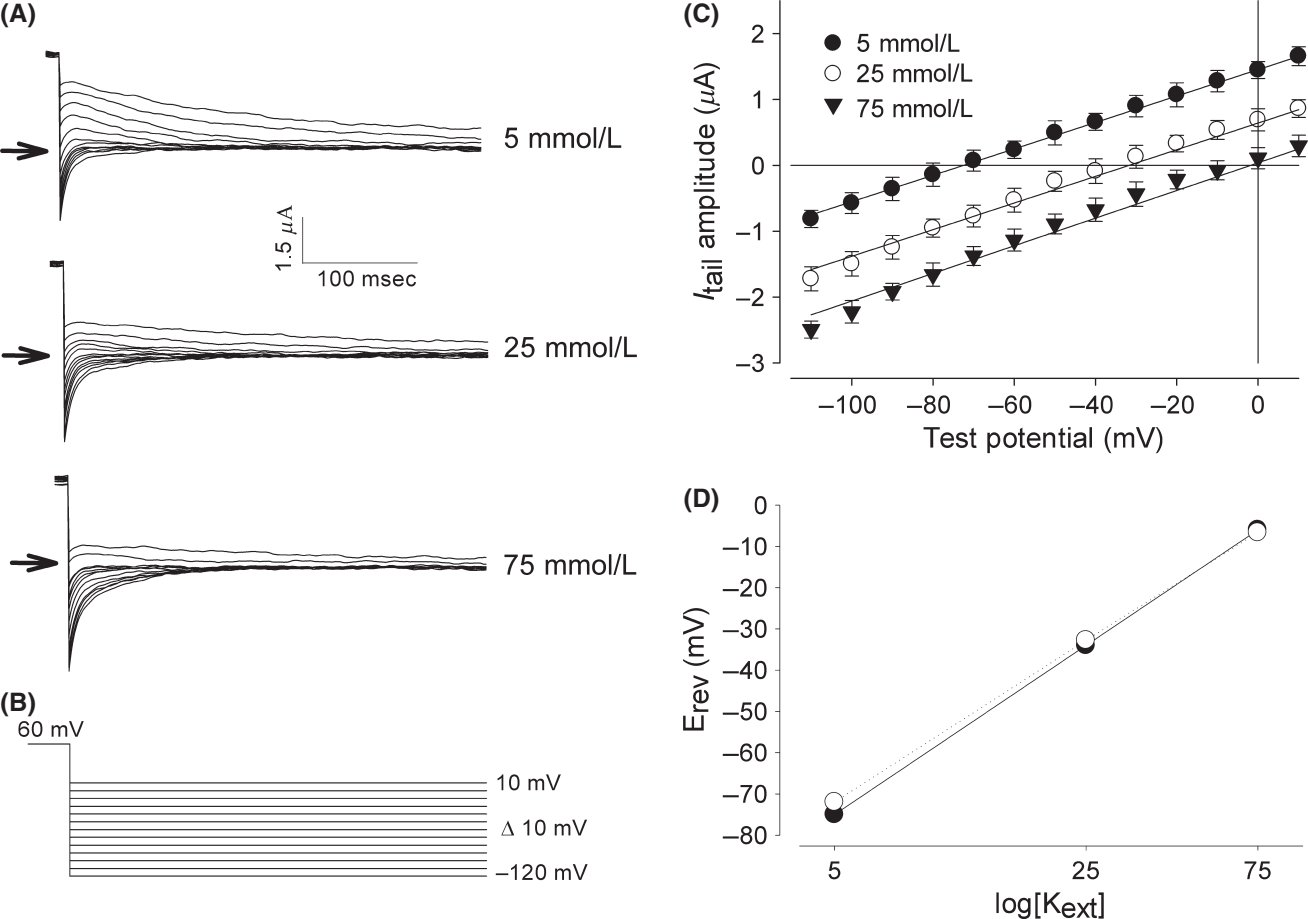
External K^+^ dependence of reversal potential. (A) Series of tail currents, at distinct external K^+^ concentrations, obtained from an oocyte injected with mRNA, in response to a stimulus protocol that, as depicted in (B), after a conditioning pulse of +60 mV, varies test voltage from −120 up to 10 mV in steps of +10 mV. (C) The relationship between the tail current amplitude and the test voltage shows a linear trend and shifts right when potassium is increased on the external media, as expected for a K^+^ selective pathway. (D) Semi‐log_10_ plot of the calculated reversal potential versus the external K^+^ concentration (empty circles) follows a linear trend with a slope of 55 mV per tenfold increase of K^+^, a value nonsignificantly distinct from the ideal value of 59 mV per tenfold (filled circles).

To verify that these currents are indeed due to K^+^ being carried across the membrane, we analyzed how their reversal potential behave in response to changes in the external K^+^ concentration. For this purpose, we made tail‐current recordings from oocytes injected with intact mRNA isolated from *G. lamblia*, on the third day after injection. Figure 3A shows a representative set of three series of tail currents from a single oocyte when external K^+^ was increased from 5 to 25 mmol/L and 75 mmol/L. Figure 3B shows the voltage protocol, consisting of a series of test pulses from −120 to +10 mV in steps of +10 mV for 0.5 sec each. Each test pulse was preceded by a conditioning pulse of 0.6 sec at +60 mV.

To estimate the reversal potential, the initial value of the deactivating (or tail) current was measured (after compensation of linear components) and plotted against the value of the test pulse and the reversal potential estimated by regression analysis. As Fig. 3C shows, increasing the external K^+^ concentration shifted the I–V relationship with the right and the reversal potential became more positive with increasing external K^+^ concentration, with values of −72 mV, −32.8 mV, and −6.6 mV for 5, 25, and 75 mmol/L of external K^+^ correspondingly. These values are closely similar to those predicted by the Nernst equation (−78.8, −37.7 and −9.7 mV), assuming an internal K^+^ of 110 mmol/L, as it has been reported elsewhere (Börjesson et al. 2011). Moreover, as Fig. 3D shows, these values show a linear trend when plotted against the log_10_ of the external K^+^ concentration with a slope of 55 mV per tenfold K^+^ increase, a value barely lower than 59 mV per tenfold K^+^ increase for a perfectly selectively K^+^ pathway according to the Nernst equation (Hille 2001). These results therefore indicate that the novel ion currents induced in oocytes by injection of *Giardia*′s mRNA are indeed due to K^+^ transmembrane flux.

### Biophysical properties of K^+^ currents induced in oocytes by injection of *Giardia lamblia* mRNA

**Figure 4.**
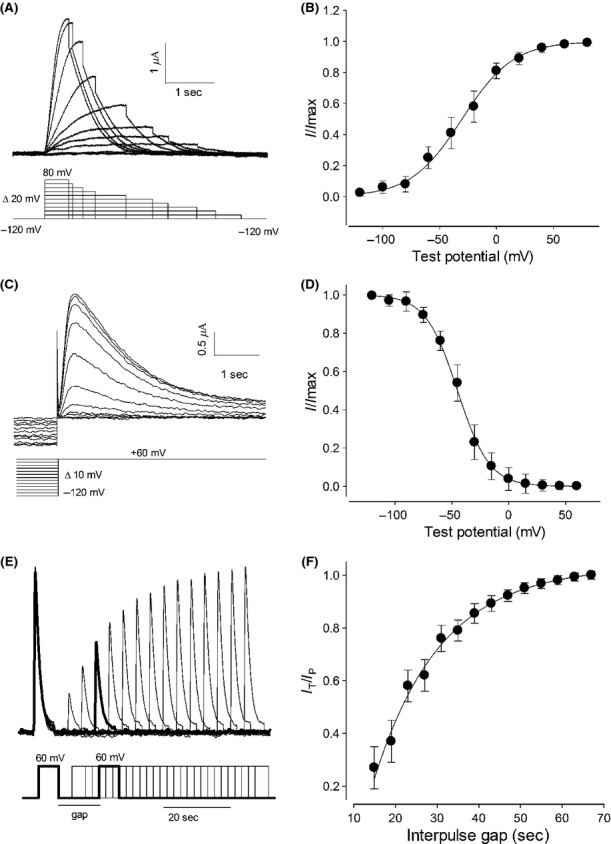
Kinetic properties of mRNA‐induced K^+^ currents. (A), (C), and (E) show in the upper part representative series of currents obtained from oocytes injected with *Giardia lamblia* mRNA in response to the voltage stimulus protocols indicated in the lower part. (A) In order to determine the voltage dependence of its activation, the instant magnitude of the tail current was measured at each test potential. (B) The standardized value of such current was averaged (25 oocytes from 3 frogs) and plotted against the test potential. (C) To determine the voltage dependence of the steady‐state inactivation, the peak value of current was measured at +60 mV after prepulses of variable voltage. (D) The mean value (23 oocytes from 3 frogs) of the standardized peak current plotted against the test voltage produces a sigmoid relationship. (E) Representative current traces from a protocol designed to determine the time recovery of inactivation. (F) Time‐dependent recovery of inactivation. To obtain this plot, the peak magnitude of the current of the second pulse was divided by the peak magnitude of the current of the first pulse and the average value of this ratio (from 18 oocytes) was plotted against the interpulse time lapse. This relationship follows an asymptotically exponential rise with a time constant of 40.5 sec.

As shown in Fig. 2A, the currents induced by intact mRNA activate in a time‐dependent manner until reaching a peak and then inactivate. The activation process gets faster as the test voltage gets more positive. The average time constant of activation at +60 mV was 110 ± 30 msec; it was obtained by fitting the raising phase of these currents to an exponential function (from 16 oocytes). Similarly, from fitting of the inactivating phase of currents at +60 mV to an exponential decay function, an average time constant of 1.92 ± 0.3 sec was obtained. As noticed, the fact that both the amplitude and the rate of activation vary with the test voltage suggests that these currents are produced by voltage‐dependent K channels, therefore our next goal was to determine their biophysical properties and to find out how these currents depend on voltage to activate and inactivate. For this purpose, ion currents were recorded from oocytes through distinct voltage protocols: First, to determine the voltage dependence of the activation, we used a protocol (as shown on Fig. 4A, lower part) consisting of series of test pulses, from −120 to +80 mV in steps of 20 mV, followed by an after pulse of −120 mV. To avoid interference of the inactivation, the length of each test voltage pulse was set to be the time required for current to reach its peak value. This protocol produced series of current traces as shown in Fig. 4A (*upper part*). From this series of current recordings we measured the amplitude of the deactivating or tail current, produced immediately after switching voltage to −120 mV, then transformed this value in a standardized form (*I*_*v*_/*I*_max_), where *I*_max_ is the maximal value obtained from such individual series. Figure 4B shows that the relationship between the average standardized magnitude of the tail current (*I*_*v*_/*I*_max_) and the test voltage follows a sigmoidal trend, therefore it was fitted to the Boltzmann equation to obtain its parametric values:
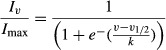


Where *I*_*v*_ is the magnitude of the tail current at a given test potential (*v*), *I*_max_ is the maximal value of current obtained from the same oocyte, V_1/2_ is the voltage at which *I*_*v*_/*I*_max_ = 0.5, and *k* is a parameter determining the steepness of the relationship. From this procedure V_1/2_ of −30.5 mV was obtained (*r*^2^ = 0.998).

To study the voltage dependence of inactivation, another pulse protocol was used. In this case, oocytes were stimulated with a series of prepulses of varying voltage levels (from −120 mV to +60 mV in steps of 10 mV), each prepulse was set for 10 sec before switching to a second pulse with a constant voltage level of +60 mV. Recording of ion currents started from the moment of switch. Figure 4C shows a representative series of such currents (*upper*) and the corresponding voltage protocol (*lower*), whereas Fig. 4D shows that the relationship between the standardized magnitude of the current at switching time and the prepulse voltage follows an inverted sigmoidal shape, with a V_1/2_ of −44.6 ± 2.4 mV (*n* = 23).

Next, to determine how these currents recover from inactivation, the protocol of stimulation consisted in series of two pulses with the same magnitude (+60 mV) and length (5 sec) but with varying interpulse gap in a range from 15 to 70 sec (Fig. 4E, *bottom*). As shown in Fig. 4E (*top*), at short interpulse intervals the peak magnitude of the current on the second pulse is notoriously reduced as compared to the current on the first pulse because of inactivation, nonetheless they recover from inactivation over time. Figure 4F shows that the ratio of the value of peak current of second pulse to the equivalent of first pulse tends to 1 as the lapse between pulses increases. This relationship follows an asymptotically exponential rise that fits with a time constant of 40.5 sec.

### Pharmacological properties of K^+^ currents induced in oocytes by injection of *Giardia lamblia* mRNA

**Figure 5.**
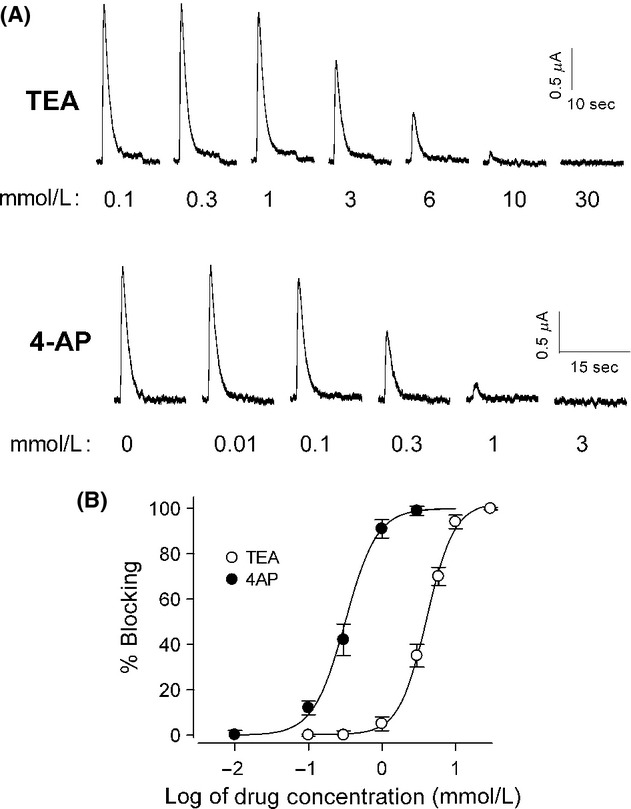
Effect of TEA and 4AP on *Giardia lamblia*′s‐mRNA‐induced K^+^ currents. (A) Representative traces showing the effect of K channel blockers TEA (*top*) and 4‐AP (*bottom*). Ion currents were elicited by a pulse of +60 mV (for 10 sec, from a holding of −80 mV) while oocytes were in a media containing drugs in increasing concentrations. From such recordings a %blocking index was calculated by referring the peak current at each drug concentration to the peak current without drugs. (B) In both cases the averaged% blocking (15 oocytes from 3 frogs each) shows a sigmoidal trend when values are plotted against the log_10_ of drug concentration.

We assayed the effect of K channel blockers TEA (tetraethyl‐ammonium) and 4‐AP (4‐aminopyridine) on *G. lamblia′s* mRNA‐induced K^+^ currents. On each case, we recorded currents in response to voltage pulses (+60 mV, 5 sec) while oocytes were immersed in external medium containing either drug at a given concentration. Figure 5A shows a representative series of current traces for TEA (*top*) and 4‐AP (*bottom*). As it can be observed, they become progressively reduced in size with increasing concentrations of any blocker, indicating that in fact such currents are sensitive to TEA and 4‐AP. To quantify their sensitivity, we calculated IC_50_, a parametric value that indicates the drug concentration producing a half blocking response. For this purpose a percent blocking index (%Bd) was obtained for each drug concentration with the following transformation:
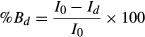


Where *I*_*d*_ is the peak value of ion currents at a given drug concentration (*d*) and *I*_0_ is the corresponding value with no drug added. As Fig. 5B shows the relationship between %Bd and log_10_ of the drug concentration follows a sigmoidal shape in both cases (4‐AP and TEA). Therefore, they were fitted to the standard Hill equation, in a modified form, as follows:
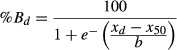


Where *x*_*d*_ is the log_10_ of a given drug concentration (*d*) and *x*_50_ is the log_10_ of IC_50_. The corresponding IC_50_ values calculated with this procedure were 0.32 mmol\L for 4‐AP and 4.04 mmol\L for TEA.

In addition to TEA and 4‐AP, which are most general K channel blockers, we probed the effect of a variety of K channel blockers, which have been reported to block distinct types of K channels. For each one of them we tested a single concentration that has been shown to effectively block K currents in other species, as charted on the IUPHAR database of voltage‐gated K channels (Chandy et al. 2013). To measure its effect we added those drugs to the external solution and compared the magnitude of the peak current at +60 mV before and after its addition. As shown in Table 1, a statistically significant reduction (*P* < 0.05, *t*‐test) was obtained by addition of the following compounds: Ba^2+^, a nonselective, low‐affinity K channel blocker (Taglialatela et al. 1993; Quinidine (200 *μ*mol\L), which preferentially blocks slow, delayed rectifier K channels (Yao et al., 1996); Quinine, a wide‐spectrum channel blocker affecting voltage‐dependent and Ca‐activated K channels (Grinstein and Foskett 1990; Kuriyama et al. 1995); Diltiazem, a Ca channel antagonist that also has been reported to block voltage‐dependent and Ca‐dependent K channels (Bukanova and Solntseva 1997); Capsaicin, the active component of chili peppers, which blocks TRPV channels as well as voltage‐dependent K channels (Kehl 1994); Charybdotoxin, which selectively blocks voltage‐dependent, Shaker‐related K channels Kv1.3 and Kv1.6 (Garcia et al. 1991); Margatoxin, which selective inhibits voltage‐dependent K channels (Kv1.3) channels (Garcia‐Calvo et al. 1993); and dendrotoxin‐I, a highly selective blocker of voltage‐gated K channels Kv1.1 and Kv1.2 (Hopkins 1998).

**Table 1.** Pharmacological properties of *Giardia lamblia*′s‐mRNA‐induced K^+^ currents. The peak current magnitude in response at +60 mV was compared before and after addition of drugs or toxins at the concentration shown on second column. A (−) sign on third column denotes nonsignificant statistical difference (*P* > 0.05, *t‐*test), (+) denotes *P* ≤ 0.05, and (++) denotes *P* < 0.005. % Block was calculated as before.

	Concentration	*P*	% Block	*n*
Ba^2+^	10 mmol/L	++	25	6
Quinine	100 *μ*mol/L	+	10	5
Quinidine	200 *μ*mol/L	++	23	6
Charybdotoxin	50 nmol/L	++	18	5
Apamin	5 *μ*mol/L	−	0	5
Dendrotoxin‐1	100 nmol/L	+	9	5
Capsaicin	50 *μ*mol/L	+	8	5
Margatoxin	10 nmol/L	++	26	5
Agitoxin	1 nmol/L	−	0	5
Glibenclamide	30 *μ*mol/L	−	0	5
Flecainide	200 *μ*mol/L	−	0	4
Diltiazem	150 *μ*mol/L	+	7	5
Kaliotoxin	50 nmol/L	−	0	4
Riluzole	70 *μ*mol/L	−	0	4

## Discussion

Ion channels had been shown to be ubiquitously present in the membrane of living cells, yet so far almost nothing is known about the variety and properties of ion channels of *G. lamblia*. To address this subject, we injected *Xenopus* oocytes with mRNA isolated from cultured *Giardia* trophozoites and found that this procedure induces expression of novel K currents which are not observed in noninjected or mock‐injected oocytes. We showed that the magnitude of K currents recorded from oocytes injected with intact mRNA increased over time after injection, whereas those from control oocytes remained unchanged.

We also analyzed its biophysical properties and found that these K^+^ currents activate in a time‐ and voltage‐dependent manner with a V_1/2_ of −30 mV and inactivate with time constant of 1.92 sec and V_1/2_ of −44.6 mV. Such properties somehow resemble those of currents produced by voltage‐dependent K channels from other species although they activate more slowly than most voltage‐dependent K currents, either A‐type or delayed rectifiers and its time constant of inactivation (1.92 sec) is larger than A‐type K^+^ currents, that typically inactivate with a time constant of 50–200 msec. Therefore, they could be more appropriately described as delayed rectifiers, although its time constant of inactivation is faster than most delayed rectifiers whose time constant of inactivation is generally larger than 4 sec.

Regarding their pharmacological properties, the fact these currents are inhibited by TEA and 4‐AP indicates that they are produced by *Giardia*′s channels that are structurally related to the voltage‐dependent K channel family; furthermore, these currents are also inhibited by quinidine, quinine, charibdotoxin, dendrotoxin‐1, capsaicin, margatoxin, and diltiazem, which are drugs and toxins reported to act more selectively on certain molecularly distinct subtypes of K channel subunits, for instance, margatoxin and charybdotoxin are common blockers of Kv1.3, whereas dendrotoxin‐1 and capsaicin are common blockers of Kv1.1 subunits; diltiazem has been reported to block Kv1.7 and Kv3.1 subunits, whereas quinidine blocks Kv1.4 and Kv1.5 and quinine blocks Kv2.2 subunits (Chandy et al.*,* 2013).

Therefore, results shown in this work indicate that the induced currents are due to the expression of Giardia′s voltage‐dependent K channels incorporated into the membrane of *Xenopus* oocytes and are the first evidence that this protozoan possesses those kinds of channels. However, we cannot determine at this stage if such currents are due to the expression of one or several distinct types of genes encoding for K channel proteins as they were produced by the injection of the full Giardia′s mRNA. Likewise we cannot rule out the possibility that there could be interaction between exogenous and endogenous K channel proteins. As the *G. lamblia* genome is already available, a further molecular characterization of the gene sequences accounting for these currents will help elucidate such possibilities.

On the other hand, we cannot determine at this stage what role these K channels play in the physiology of Giardia′s trophozoites. However, from what has been learned from other species, we can speculate that they could be involved in any of several processes including membrane potential regulation, membrane excitability, cell motility, and cell volume regulation among others. In support of some of this possibilities, there are reports indicating that potassium flux plays a role in the response of *Giardia* to hyposmotic challenges (Maroulis et al. 2000) and in regulating the resting membrane potential (Biagini et al. 2000).

Finally, besides of showing evidence of *Giardia* K channels, the findings of this work could be of therapeutical relevance as these channels could be potential targets to develop drugs that could be used for giardiasis if they happen to be crucial for the protozoan survival.

## Conflict of Interest

None declared.
